# PCDTBT: Force Field Parameterization and Properties by Molecular Dynamics Simulation

**DOI:** 10.1021/acs.jpcb.4c08393

**Published:** 2025-03-21

**Authors:** Konstantinos Kordos, Konstantinos Kaklamanis, Maria Andrea, Dimitrios G. Papageorgiou

**Affiliations:** Department of Materials Science and Engineering, University of Ioannina, POB 1186, Ioannina GR45110, Greece

## Abstract

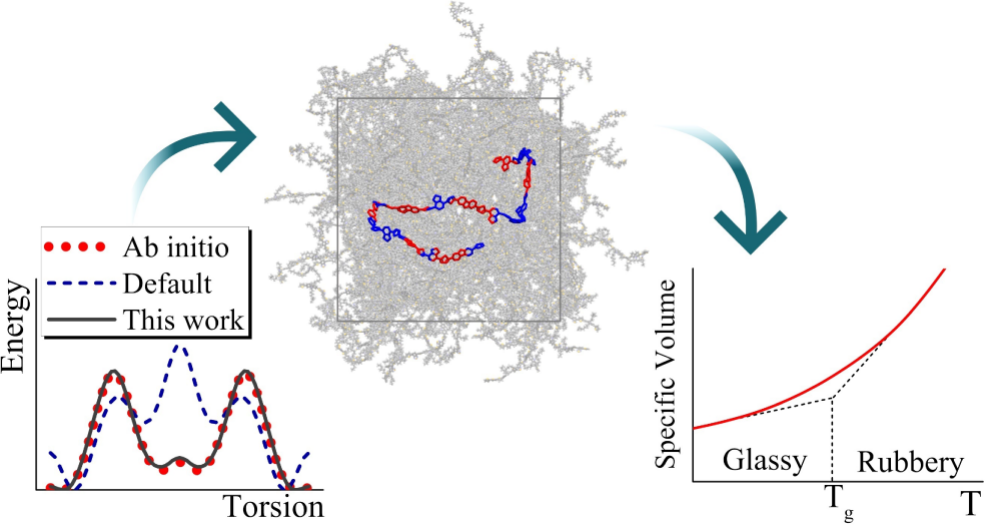

Conjugated polymers are indispensable building blocks in a variety of organic electronics applications such as solar cells, light-emitting diodes, and field-effect transistors. Poly[*N*-9′-heptadecanyl-2,7-carbazole-*alt*-5,5-(4′,7′-di-2-thienyl-2′,1′,3′-benzothiadiazole)] (PCDTBT) is a carbazole-benzothiadiazole-based copolymer with a donor–acceptor structure, consisting of electron-donating and electron-withdrawing subunits and featuring a low band gap. In this work, the General Amber Force Field is extended in two ways, specifically for modeling PCDTBT. First, a set of partial atomic charges is derived that mimic a long chain and adequately describe different conformations that may be encountered in a bulk environment. Second, torsional terms are reparametrized for all dihedral angles in the backbone via ab initio computations. Subsequently, a series of large-scale Molecular Dynamics simulations are employed to construct and equilibrate bulk ensembles of three PCDTBT oligomers using different starting conformations of the oligomer chains. Several structural properties are computed, namely mass density, chain stiffness (through persistence length and Kuhn segment length), and glass transition temperature. Our results are in good agreement with available literature data, demonstrating the suitability of the new parametrization.

## 1 Introduction

Conjugated polymers (CPs) are being intensely investigated, both experimentally and theoretically, due to their exceptional optoelectronic properties, giving rise to unique applications in the fields of organic photovoltaics (OPVs),^1−3^ organic light emitting diodes (OLEDs),^4−6^ organic field effect transistors (OFETs)^7,8^ and organic sensors.^9−11^ In these applications CPs deliver major advantages such as mechanical flexibility, lightweight, semi transparency, low-cost, and manufacturing via scalable processes such as roll-to-roll printing. CPs usually consist of a π-conjugated backbone where side chains are added to increase solubility in various solvents and aid device fabrication. Conjugated homopolymers include polythiophenes, polypyrroles, polyfluorenes and their various modifications.^12,13^

Charge transport in these materials proceeds by thermally activated hopping between localized sites, facilitated by overlapping orbitals between neighboring chains.^14,15^ Close relation between morphology and optoelectronic properties is by now well-established, and many computational investigations exist in the literature employing classical Molecular Dynamics and ab initio calculations to study and elucidate charge transport mechanisms and related properties in a variety of polymers such as P3HT,^16−23^ PBTT,^23−25^ IDTBT,^26^ TIFBT,^26^ MEH-PPV,^27^ PTMA,^28^ PCPDTBT,^29^ TFB,^30^ PDPPTT-T-10^31^ and CYTOP.^32^ In all these cases, Molecular Dynamics simulation is used to obtain realistic CP morphologies, with the underlying force field being of paramount importance since it directly influences the results. Wolf et al.^33^ used P3HT as a model system and investigated the effect of various force field parameters in Molecular Dynamics (MD) simulations, deducing that partial atomic charges and torsion potentials have the greatest impact on structure and dynamics related to charge transport mechanisms in P3HT.

Many general-purpose force fields exist in the literature, such as OPLS,^34^ CGenFF,^35^ GAFF^36^ and GROMOS,^37−39^ demonstrating successful predictions in organic compounds. However, it is well-known^40^ that in most cases their description of a CP is rather poor since the torsional potentials along the backbone are heavily affected by conjugation. Usually, energy barriers and dihedral angle equilibrium positions are in error. Hence, an accurate FF that is able to describe correctly the CP is a prerequisite to any effective MD simulation. In several cases these general-purpose FFs have been reparametrized to treat conjugated homopolymers. Marcon and Raos were the first to derive an improved set of parameters for oligothiophenes^41^ and oligofluorenes^42^ built on the MM3^43−45^ force field. The OPLS all-atom force field was reparametrized for polythiophene,^46,47^ polyfluorene^46^ and poly(alkylthiophene).^48,49^ The widely used P3HT nowadays is considered a model semiconducting polymer and thus has received considerable attention, with several authors^19,22,47,48,50−52^ deriving parameters for this CP. Some of these specific parametrizations for P3HT have been comparatively tested with varying degrees of success.^33,53^ Other reparameterizations for MEH-PPV,^54^ isoindigo-thienothiophene,^55^ diketopyrrolopyrroles,^56^ PEDOT^57^ and other polymers also exist in the literature.

To tune energy levels and reduce the bandgap in a CP, the donor–acceptor (DA) structure has been devised,^58−64^ where electron-rich (donor) and electron-deficient (acceptor) units are combined along the CP backbone. Common donors include thiophene-based ring systems (either fused or bridged), while acceptors typically contain electron withdrawing moieties such as ketone, thiadiazole, and halogenated groups. Although force field reparameterizations for such copolymers are scarce in the literature, Jackson et al.^65^ developed new force field parameters based on OPLS, for 15 low bandgap conjugated copolymers commonly used in OPV and OFET applications.

Polymer poly[*N*-9′-heptadecanyl-2,7-carbazole-*alt*-5,5-(4′,7′-di-2-thienyl-2′,1′,3′-benzothiadiazole)], also known as PCDTBT is a donor–acceptor copolymer with an electron rich carbazole unit and an electron deficient benzothiadiazole unit, linked by thienyl bridges. PCDTBT can be produced by low-cost, facile, reliable, and scalable synthetic procedures^66^ and is combined with the PC70BM electron acceptor in OPV bulk heterojunction architectures, yielding power conversion efficiency (PCE) up to 7.5%, with an estimated lifetime up to 7 years.^66,67^ To increase PCE, PCDTBT has been investigated experimentally in conjunction with gallium porphyrins,^68^ graphene nanoplatelets,^69^ fluorinated multiwalled carbon nanotubes,^70^ semitransparent quasi-heterojunction active layer structures,^71^ nonfullerene acceptor based OPVs,^72^ aqueous dispersions of PCDTBT:PC70BM nanoparticles^73^ and colloidal Cu_2_ZnSnS_4_ nanocrystals.^74^ Various structural, optical and thermal properties have also been examined.^75^ Computational studies for PCDTBT are also present in the literature. Kawanabe et al.^76^ using Molecular Dynamics simulations examined the atomic scale structure and structural order of PCDTBT in toluene solvent. Franco^77^ investigated the structural and optoelectronic properties of several substituted PCDTBT oligomers by DFT and TD-DFT, while Van den Brande et al.^78^ also using TD-DFT examined the singlet excitation schemes of PCDTBT at the interface with PCBM. A hierarchical multiscale method was applied by Li and Lagowski^79^ to investigate charge transport in ordered and orientationally disordered PCDTBT.

In this work we present a reparameterization of the fully atomistic General Amber Force Field (GAFF) for accurately modeling PCDTBT. To this end, we applied ab initio computations to reparametrize all dihedral angles in the PCDTBT backbone and create a set of partial atomic charges that mimic a long chain. Subsequently a series of large-scale Molecular Dynamics simulations were performed for different oligomers and starting conformations of PCDTBT to demonstrate the new force field with the calculation of several structural properties such as persistence length, Kuhn segment length, and glass transition temperature. Our results are then compared with literature data where available.

## 2 Methods

### 2.1 Force Field Modeling

Our PCDTBT modeling is based on the General Amber Force Field (GAFF)^36^ where the total potential energy of a system is expressed as a sum of interactions between bonded and nonbonded atoms: *E*_*tot*_ = *E*_*b*_*+E*_*nb*_. The former includes terms for bond stretching, angle bending and dihedral angle rotations. They are given by
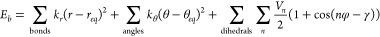
1with *k*_*r*_, *k*_θ_ being the force constants for bond stretching and angle bending, and *r*_*eq*_, θ_*eq*_ the corresponding equilibrium distances. Torsional terms are described by a truncated Fourier series with *V*_*n*_, *n*, γ being the amplitude, multiplicity, and phase correspondingly. Nonbonded interactions include electrostatic and van der Waals terms
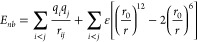
2where q*_i_* are partial atomic charges and *r*_0_, *ε* are the equilibrium distance and potential depth in the Lennard-Jones function.

### 2.2 Torsional Parameters

**Figure 1:**
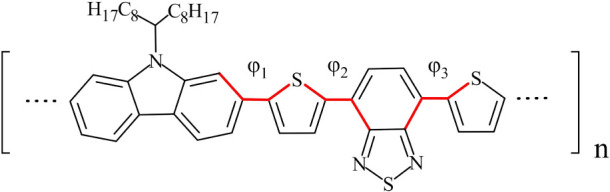
PCDTBT structural repeat unit. Backbone dihedrals φ_1_,φ_2_,φ_3_ are shown in red.

Figure 1 depicts the basic structural repeat unit, also referred to in the following as a PCDTBT monomer, along with the three dihedral angles that characterize the backbone. To derive the ab initio torsional profiles for dihedrals φ_1_, φ_2_, φ_3_ a simpler moiety was used. Starting from a single PCDTBT repeat unit, side chains were removed, and bonds were saturated with hydrogen atoms. The resulting structure is depicted in Figure S1. A full geometry optimization was performed using Density Functional Theory with the long-range corrected LC-ωPBE^80^ exchange and correlation functional and the 6–31G(d,p) basis set. Long-range corrected functionals are known to reduce the many-electron self-interaction error leading to an improved description of torsion barrier heights.^81^ The optimized structure was used as a starting point and a series of partial geometry optimizations were performed, keeping the dihedral angle of interest fixed at a specific value, while all other degrees of freedom were allowed to relax. The procedure was repeated in 5° increments for each of the dihedral angles φ_1_, φ_2_, φ_3_.

**Figure 2:**
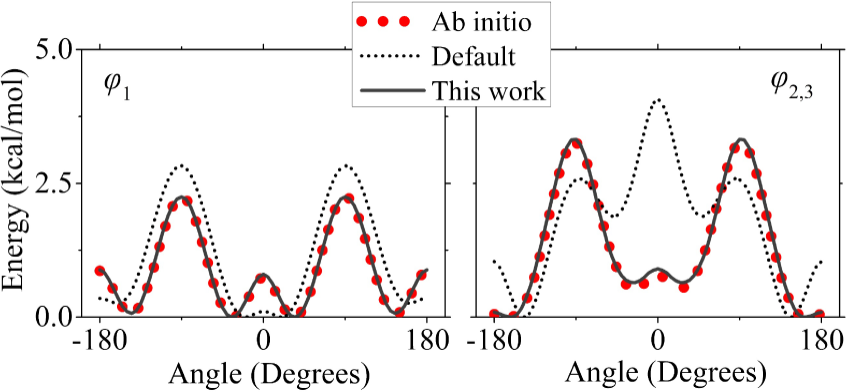
Ab Initio torsional energy profiles (red dots) along with the default GAFF force field (black dots) for φ_1_ (left panel) and φ_2,3_ (right panel). Solid black lines represent the new parametrization.

Torsional profiles for dihedral angles φ_1_, φ_2_, φ_3_ were also derived using the default GAFF parameters for a PCDTBT monomer and are depicted in Figure 2 along with the ab initio profiles. Due to symmetry, torsional profiles for φ_2_ and φ_3_ were averaged and denoted as φ_2,3_. It is evident that neither the position of the minima nor the corresponding barrier heights are reproduced correctly with respect to the ab initio data, with dihedral φ_2,3_ exhibiting the largest discrepancy.

Our strategy is to adjust the torsional terms *V*_*n*_ for dihedrals φ_1_ and φ_2,3_, so that the total potential energy from the force field matches the corresponding ab initio value. For this purpose, the total potential energy is written as *E*_*tot*_(φ)=*E*_0_(φ)+*V*(φ), where *V*(φ) is the torsional term and φ stands for φ_1_ or φ_2,3_. Contribution *E*_0_(φ) represents all energy terms besides *V*(φ), i.e., bond stretching, angle bending, VdW and electrostatic interactions, and contributions from all other dihedral angles. *E*_0_(φ) was calculated by setting to zero the appropriate torsional terms for dihedral φ, and performing a partial geometry optimization while keeping φ fixed at the value of interest. The procedure was repeated in 5° increments. After subtracting from the ab initio profile, the torsional potential *V*(φ)=*E*_*tot*_(φ)-*E*_0_(φ) was obtained by least-squares fitting to a truncated Fourier series of the form
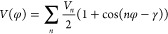
3setting the phase γ to 180°. The fit was performed utilizing terms up to *V*_4_ for φ_1_ and *V*_6_ for φ_2,3_, which is a good compromise between accuracy and computational efficiency. *V*_3_ and *V*_5_ were set to zero due to symmetry. The resulting parameters are listed in Table 1. The new profiles are also depicted in Figure 2 along with the ab initio values for comparison. Ab initio torsional profiles were derived using the NWChem^82^ software package.

**Table 1:** Derived Torsional Parameters for Dihedrals φ_1_ and φ_2,3_ (in kcal/mol)

	V_1_	V_2_	V_4_	V_6_
φ_1_	–0.0429	2.2460	–0.2588	
φ_2,3_	1.0358	4.0936	0.2693	0.1345

### 2.3 Partial Atomic Charges

In order to maintain compatibility with the overall GAFF parametrization procedure,^36^ the electrostatic potential was derived at the HF/6–31G(d,p) level, and the RESP scheme^83^ was used to fit the partial atomic charges. Our design philosophy has two goals. First, to define a set of partial charges that mimic a long (infinite) chain and second, to provide a set of charges that adequately describes different configurations that may be encountered in successive monomers in the polymer chain. Ideally, the charge derivation procedure should be applied in a chain with increasing number of monomers. Then, charges of a monomer in the middle of the chain should be examined until converge is achieved. This procedure accurately determines the required partial charges and has been applied in the past in cases where the monomer unit is small.^46^ Nevertheless, given the size of the PCDTBT repeat unit, ab initio computations on chains with a large number of monomers are quite demanding or even prohibitive.

To overcome this obstacle, we decomposed the PCDTBT monomer into a sequence of four subunits: carbazole, thiophene, benzothiadiazole and thiophene denoted as C, T, B, T correspondingly. We adopt the convention that each PCDTBT chain must be terminated at both ends by a thiophene (T) subunit. Hence, in our force field model five different units were used: C_i_, B_i_, T_i_ (inside the chain), *T*_c_ (at the end of the chain, adjacent to C) and T_b_ (at the end of the chain, adjacent to B). Two dimer chains (denoted as chain A and chain B) were created with different subunit ordering. Chain A is constructed by the sequence CTBT_i_C_i_T_i_BT_b_ while chain B by BTCT_i_ B_i_T_i_C*T*_c_. Note that in these sequences, unit C_i_ is approximately at the middle of chain A, while B_i_ is at the middle of chain B. Two configurations were created per chain. One where the two monomers of the dimer have the carbazole side chains in the same direction (Z configuration), and one with side chains in the opposite direction (E configuration). See Section 2.4 for a description of E and Z configurations. The geometry of all four configurations was optimized at the LC-ωPBE/6–31G(d,p) level and the electrostatic potential was then extracted by a single point computation at the HF/6–31G(d) level. For each chain, partial atomic charges were fitted simultaneously for both configurations E and Z by the RESP method.

**Figure 3:**
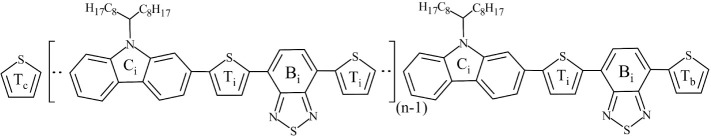
PCDTBT model constructed from individual subunits. Partial atomic charges for each subunit are provided in Supporting Information.

Using the derived partial charges for the five units, model polymer chains with n repetitions of the monomer are constructed (Figure 3) as *T*_c_ (C_i_T_i_B_i_T_i_)_n-1_···C_i_T_i_B_i_T_b_, that is, partial atomic charges for the C and B units are obtained from the corresponding units in the middle of chains A and B, i.e., C_i_ and B_i_. Charges for T units are averaged between the four T_i_ units in both chains, while charges for the terminal units *T*_c_ and T_b_ were taken from chains B and A correspondingly. Charges were normalized and are provided in Tables S1–S6. All ab initio computations for the derivation of partial charges were performed by the Gaussian^84^ software package.

### 2.4 Molecular Dynamics Simulations

**Figure 4:**
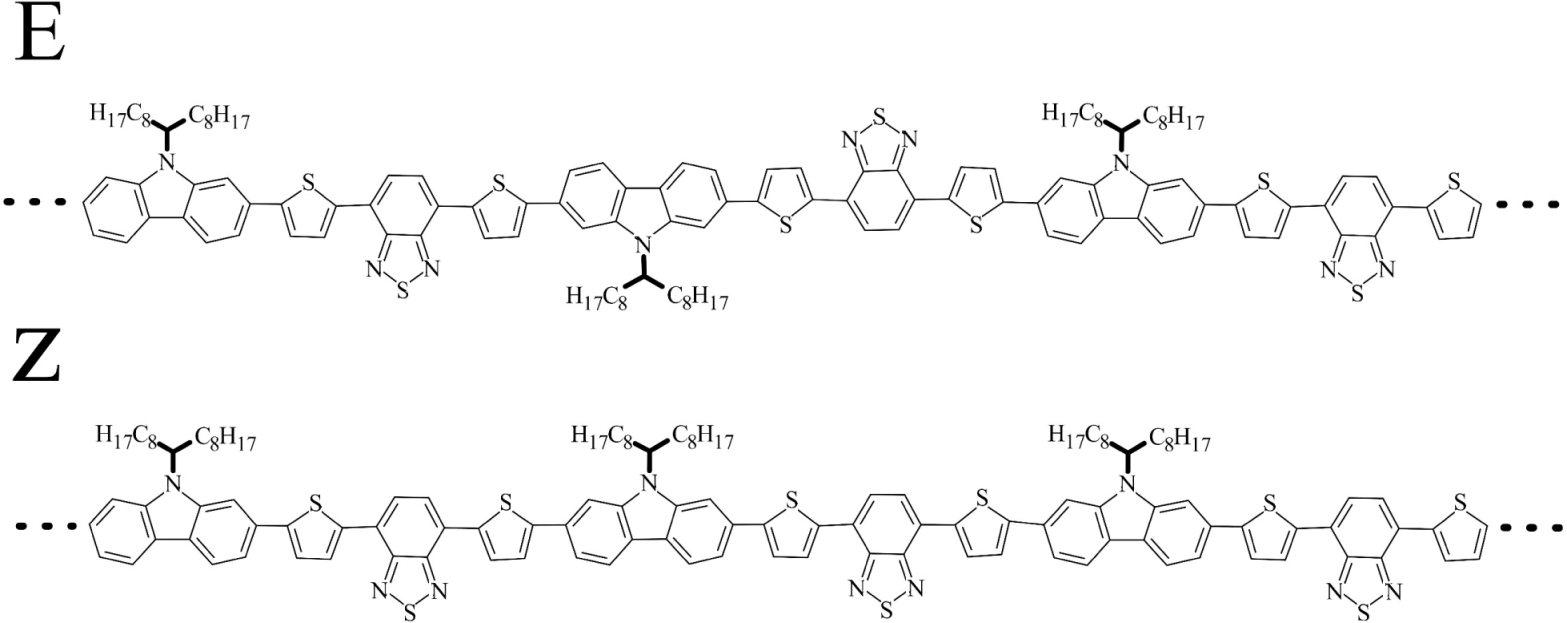
Schematic representation of PCDTBT oligomers with carbazole side chains pointing in opposite directions (E) or in the same direction (Z).

Molecular Dynamics simulations were performed for bulk systems under periodic boundary conditions consisting of PCDTBT oligomers with 6, 8, or 16 repeat units. These are denoted as P6, P8 and P16. For each of the first two cases, two different starting conformations were used by adjusting dihedral angle φ_1_ between successive monomers, as sketched in Figure 4. Setting dihedral angle φ_1_ between two successive monomers to 40° results in a configuration with carbazole side chains pointing to the same direction (denoted as configuration Z). Setting dihedral angle φ_1_ between two successive monomers to 150° results in a configuration with carbazole side chains pointing to opposite directions (denoted as configuration E). In the case of P16, only an E configuration was used. Hence, a total of five different bulk systems were considered, namely P6E, P6Z, P8E, P8Z and P16E.

Initial bulk configurations were prepared using PACKMOL^85^ by randomly placing 167 PCDTBT hexamers or 125 octamers or 25 16mers in a cubic box at low density, ensuring that there are no overlapping atoms. After a short minimization session to relax close contacts, the five configurations were equilibrated in the isothermal–isobaric (NPT) ensemble at a high temperature to allow the systems to overcome barriers and reach structural equilibrium. We have chosen 1100 K, which is approximately the highest temperature, where the systems retain their liquid structure. Equilibration times were 60, 135, and 780 ns for P6, P8 and P16 respectively. Measurements were recorded during additional trajectories of 15 ns (P6), 20 ns (P8) and 120 ns (P16). Using the same protocol a separate P8Z system was also run with the default GAFF parametrization for comparison. All Molecular Dynamics simulations were performed using NAMD^86^ at a pressure of 1 atm, using a time step of 1 fs. Long range electrostatic interactions were treated with the PME method^87^ and a grid size of 1 Å. Non bonded interactions were cut off at 10 Å, while a switching function was enabled at 9 Å. Bonds between heavy atoms and hydrogen were fixed at their equilibrium values using the SHAKE algorithm.

## 3 Results and Discussion

### 3.1 Microscopic Characteristics

#### 3.1.1 Structural Equilibration

**Figure 5:**
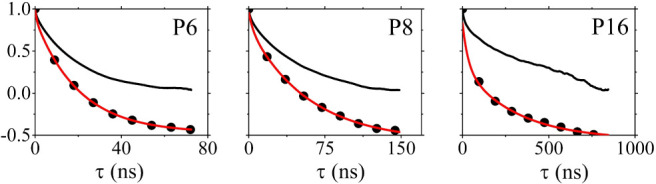
End-to-end time autocorrelation functions (TACF) (black solid lines), *P*_2_(TACF(τ)) (black dots) and exponential fitting (red solid lines) for P6, P8 and P16 at *T* = 1100 K.

To ensure that our simulations for the five bulk PCDTBT systems have run long enough to obtain adequately equilibrated structures we calculated *P*_2_(TACF(τ)), where *P*_2_(*x*)=(3*x*^2^–1)/2 is the second order Legendre polynomial, and TACF(τ) is the end-to-end time autocorrelation function, defined as
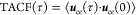
4***u****_ee_*(τ) is a unit vector along the line connecting the two ends of the PCDTBT oligomer chain. *P*_2_(TACF (τ)) are depicted in Figure 5 and clearly indicate two processes with different relaxation times. These were identified by fitting *P*_2_(TACF(τ)) to a sum of two exponentials as
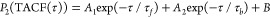
5where *A*_1_, *A*_2_ and *B* are constants. Characteristic time τ_*f*_ corresponds to a fast process due to the relaxation of dihedral potentials around their minimum values, while τ_*b*_ corresponds to a slow process where backbone dihedrals transition between their equilibrium values, crossing the corresponding energy barriers. The calculated relaxation times are listed Table 2. We note that the backbone relaxation time τ_*b*_ as well as all other calculated quantities in our simulations show no significant dependence on the initial conformation (E or Z), hence in what follows, only averages between E and Z configurations are reported, while detailed measurements are provided in Supporting Information. Statistical error bars were calculated for Figure 5 and all subsequent MD related figures by the block average method.^88^ They were found to be smaller than the size of the corresponding symbols and were omitted for clarity.

**Table 2:** *P*_2_(TACF(τ)) Relaxation Times (in ns) for P6, P8, and P16 at *T* = 1100 K

	τ_*f*_	τ_*b*_
P6	0.98	10.72
P8	2.50	25.27
P16	28.69	287.20

**Figure 6:**
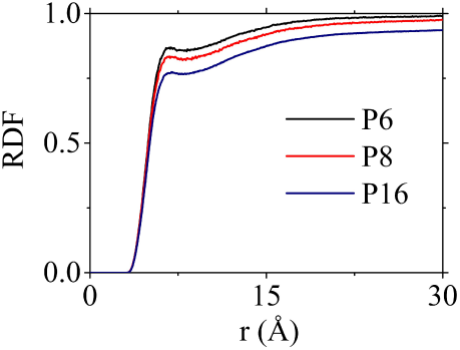
Total radial distribution function (RDF) for P6, P8 and P16 at *T* = 1100 K.

To verify the amorphous structure of the five oligomer systems we calculated the total intermolecular radial distribution function (RDF) between the centers of mass of all four PCDTBT subunits (C,T,B,T) (Figure 6). For the carbazole subunit only atoms on the oligomer backbone were considered, omitting side chains. The total RDF exhibits the typical behavior of an amorphous system, without any indications of molecular ordering or crystal packing, as expected for this elevated temperature. In addition, partial RDFs were also calculated for the C, B and T subunits and are provided in (Figure S3).

#### 3.1.2 Chain Stiffness

**Figure 7:**
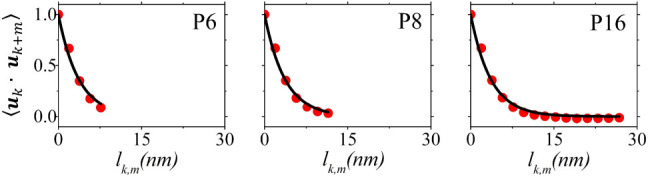
Autocorrelation function ⟨***u****_k_****u****_k+m_*⟩ as a function of *l*_*k,m*_ (red dots) and the corresponding exponential fit (black solid lines) for P6, P8 and P16.

Persistence length and Kuhn segment length are important structural metrics that characterize the stiffness of the polymer chain.^89^ They can be measured experimentally using Atomic Force Microscopy,^90−92^ viscosity experiments^93,94^ and Small Angle Neutron Scattering.^95−97^ Persistence length has been shown recently^98^ to correlate with the optical absorption of various polymer materials. Persistence length was determined by calculating the autocorrelation of vectors tangent to monomers and fitting the result to an exponential decay function
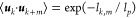
6In this relation *l*_*p*_ is the persistence length, ***u****_κ_*, ***u****_κ+m_* are unit vectors tangent to monomers *k*, *k* + *m* respectively, and *l*_*k,m*_ is the contour length from monomer *k* to monomer *k* + *m*. Figure 7 depicts the vector autocorrelation function ⟨***u****_k_****u****_k+m_*⟩ as a function of *l*_*k,m*_ and the corresponding fit. Kuhn length was calculated as
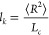
7where ⟨*R*^2^⟩ is the mean square end-to-end distance on the polymer backbone chain and *L*_*c*_ is the respective contour length of the whole chain.

**Figure 8:**
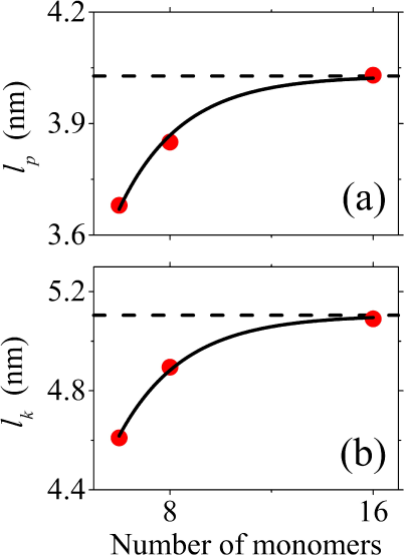
Extrapolation of (a) persistence length (*l*_*p*_) and (b) Kuhn length (*l*_*k*_). Red dots stand for the calculated values, taken from Table 3. Black solid lines represent the fitting function as described in the text. Black dashed lines indicate the infinite limit.

Table 3 lists the calculated *l*_*p*_ and *l*_*k*_ at *T* = 1100 K and *p* = 1 atm using ensemble and time averages for P6, P8 and P16. It is observed that both lengths increase with the number of PCDTBT monomers *n*. Fitting the calculated *l*_*p*_ and *l*_*k*_ values with the exponential function *l*(*n*)=*l*_∞_(1–exp(−*n*/*b*)) (with *b* being a constant) yields the corresponding predictions for the infinite chain as *l*_*p*∞_ = 4.028 nm and *l*_*k*∞_ = 5.105 nm (Figure 8). The calculated *l*_*p*_ and *l*_*k*_ for P16 are 4.03 and 5.09 nm, differing by less than 0.05% and 0.3% from their predicted infinite chain values *l*_*p*∞_ and *l*_*k*∞_ correspondingly, indicating that the P16 oligomer has captured the behavior of a long chain. In addition, both the calculated *l*_*p*_ and *l*_*k*_ for P8 differ by less than 4% from their predicted infinite chain values *l*_*p*∞_ and *l*_*k*∞_, while for P6 the differences are less than 10%. This suggests that even the small oligomer models are suitable for estimating these quantities as a computationally affordable alternative. Table 3 also lists the results from the P8Z system with the default GAFF dihedral angle parameters. Both *l*_*p*_ and *l*_*k*_ are overestimated by the default parametrization by 19% and 22% correspondingly.

**Table 3:** Persistence Length (*l*_*p*_) and Kuhn Length (*l*_*k*_) (in nm) for P6, P8 and P16 at *T* = 1100 K

	*l*_*p*_	*l*_*k*_
P6	3.68	4.61
P8	3.85	4.90
P16	4.03	5.09
Extrapolated	4.03	5.11
Default GAFF	4.60	6.00

Although experimental persistence length and Kuhn segment length measurements for homopolymers are readily available,^99,100^ such measurements have not been reported for PCDTBT. Persistence length for a structurally similar polymer, PFTBT, has been estimated as 5.9 nm by Zhang et al.^101^ using the hindered rotation (HR) model^102,103^ and employing DFT computations. However, as reported by the authors, when the HR model is applied to P3HT it overestimates the experimental persistence length by 33%. We note that PFTBT is similar to PCDTBT in the sense that there is a fluorene unit instead of carbazole. In another similar donor–acceptor copolymer, van der Scheer et al.^104^ estimated the effective persistence length of nonstoichiometric poly(dioctylfluorene-*alt*-benzothiadiazole) (F8BT) doped with dithienyl benzothiadiazole (DTBT) to be approximately 4 nm. Kuhn segment length for the same copolymer was estimated as 6–8 nm.^105^

Persistence length and Kuhn segment length were also estimated at two lower temperatures, namely 900 and 700 K. At even lower temperatures, near the glass transition (see Section 3.2) structural equilibration may be impossible to perform at reasonable simulation times, since there is not enough thermal energy to drive the system to equilibrium. In this case only the P8Z system was quenched to 900 K and then to 700 K with a rate of 5 K/ns, followed by simulation in the NPT ensemble for a total of 150 and 250 ns correspondingly. The calculated properties at these two temperatures are listed in Table 4. Both *l*_*p*_ and *l*_*k*_ were found to have an inverse dependence on temperature. The same inverse behavior has been observed for the thiophene based polymers P3HT,^53,106^ P3EHT,^106^ and the fluorene-based polymer PFO.^106^

**Table 4:** Persistence Length (*l*_*p*_) and Kuhn Length (*l*_*k*_) (in nm) for P8Z at *T* = 900 and 700 K

T (K)	*l*_*p*_	*l*_*k*_
900	3.92	5.18
700	3.94	5.34

### 3.2 Glass Transition Temperature

**Figure 9:**
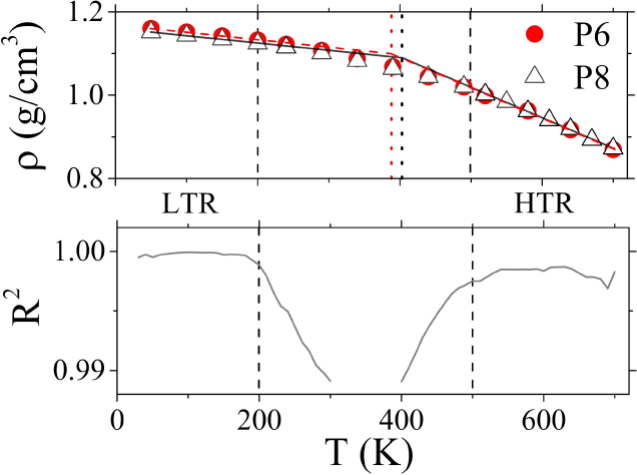
Mass density (upper panel) and coefficient of determination (lower panel) as a function of temperature for P6 and P8. Vertical dashed lines distinguish the low (LTR) and high (HTR) temperature regions. Vertical dotted lines in the upper panel indicate *T*_g_ for P6 (388 K) and P8 (403 K).

The glass transition temperature (*T*_g_) characterizes the thermal stability of the material and consequently serves as an indicator of its lifetime.^107−110^ It can be measured experimentally using Differential Scanning Calorimetry,^111^ Thermal Mechanical Analysis^112^ or Dynamic Mechanical Analysis.^113^ To extract *T*_g_ the initial melt was slowly cooled down while the density of the system was monitored (Figure 9). Two distinct linear regions appear in the density–temperature plot, one at high (HTR) and one at low temperatures (LTR). The two regions were fit by straight lines, the intersection of which gives an estimate for *T*_g_. To define the limits for each region, we used a sliding window and calculated the coefficient of determination (*R*^2^) for the linear fit using the points within the window. Linearity in the density–temperature plot is indicated by a plateau in the corresponding *R*^2^ value (at *R*^2^≈1), while a change of slope in the density–temperature plot is signaled by a steep drop in *R*^2^, thus providing limits for the linear fit in the corresponding region.

Since we already established that previous results show no significant dependence on the initial E or Z structures, this procedure was applied to P6E and P8Z using a cooling rate of 5 K/ns. The results are depicted in Figure 9 along with the *R*^2^ coefficient and the LTR, HTR linear regions. The resulting *T*_g_ is 388 K for P6E and 403 K for P8Z. Experimental *T*_g_ measurements available in the literature are in the range 383–402 K.^114,115^ In addition, during the cooling process the mass density at 300 K was recorded as 1.10 g/cm^3^ while available experimental measurements at the same temperature are in the range 1.13–1.16 g/cm^3^.^116−118^

## 4 Conclusions

In summary, we developed a set of parameters for the PCDTBT polymer, based on the all-atom General Amber Force Field force field. We derived a set of partial charges using the RESP scheme and potentials for the backbone dihedral angles using ab initio calculations. The methodology can be easily applied to other conjugated copolymers as well. Subsequently, a series of large-scale simulations were performed using different PCDTBT oligomers. To enhance statistical sampling, different starting conformations of the oligomer chains were also used. Calculated chain stiffness, mass density and glass transition temperature are in good agreement with available literature data. By performing simulations with the default GAFF force field, we demonstrate the improved behavior of the new parametrization. Finally, based on the different systems studied, we suggest that an oligomer with eight structural repeat units may be used as a representative yet computationally cost-effective model for PCDTBT. Applications for the new parametrization include further studies of PCDTBT properties in various environments, exploration of structure–property relationships, especially in the context of charge or exciton transport, and development of coarse models for PCDTBT, thus enabling investigations with larger systems and longer time scales.

## Supporting Information Available

Derived atomic partial charges and detailed measurements for all PCDTBT oligomers (PDF)
Atomic coordinates for a PCDTBT structural repeat unit and force field parameters (ZIP)
