# Supplementary material for: PCDTBT: Force Field Parameterization and Properties by Molecular Dynamics Simulation
Source: J Phys Chem B. 2025 Mar 21;129(13):3492–501. doi: 10.1021/acs.jpcb.4c08393 (PMC11973877; doi:10.1021/acs.jpcb.4c08393)
Supplement: Supplementary file 1 — jp4c08393_si_001.pdf [file jp4c08393_si_001.pdf]

PCDTBT: Force Field Parameterization and Properties by Molecular Dynamics Simulation

Supporting Information

Konstantinos Kordos, Konstantinos Kaklamanis, Maria Andrea, Dimitrios G. Papageorgiou*

Department of Materials Science and Engineering

University of Ioannina

POB 1186, GR 45110, Ioannina, Greece

* Corresponding author. E-mail address: dpapageo@uoi.gr

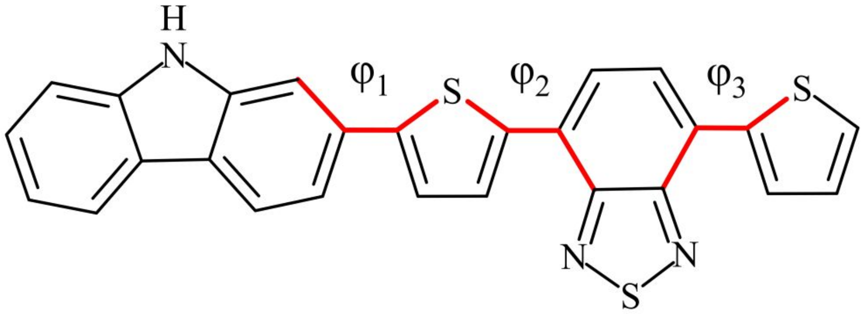

Figure S1. Simpler moiety used to derive the ab initio torsional profiles.

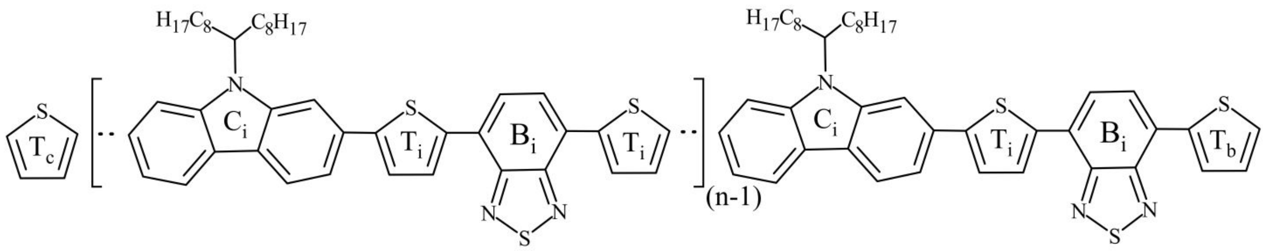

Figure S2: PCDTBT model constructed from individual subunits.

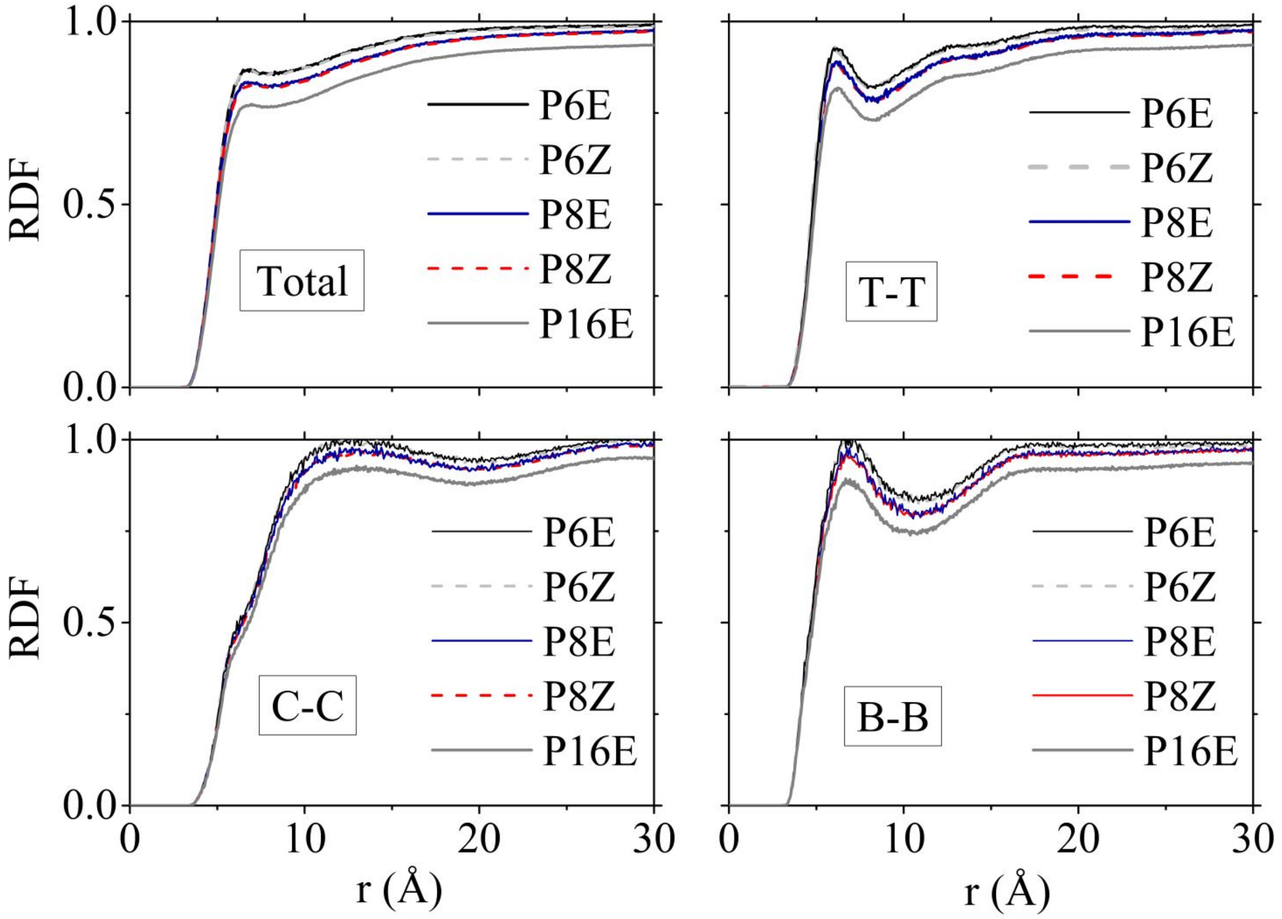

Figure S3: Total and partial radial distribution functions for all PCDTBT chain conformations at T=1100K.

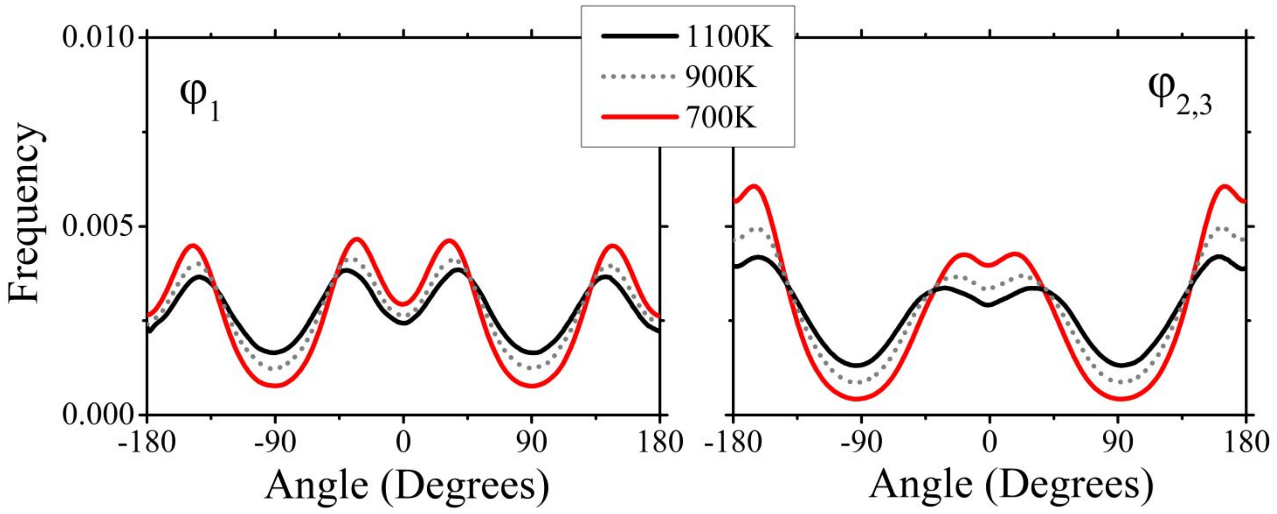

Figure S4: Dihedral angle histograms for φ_1 and $\varphi_{2,3}$ at the three temperatures studied.

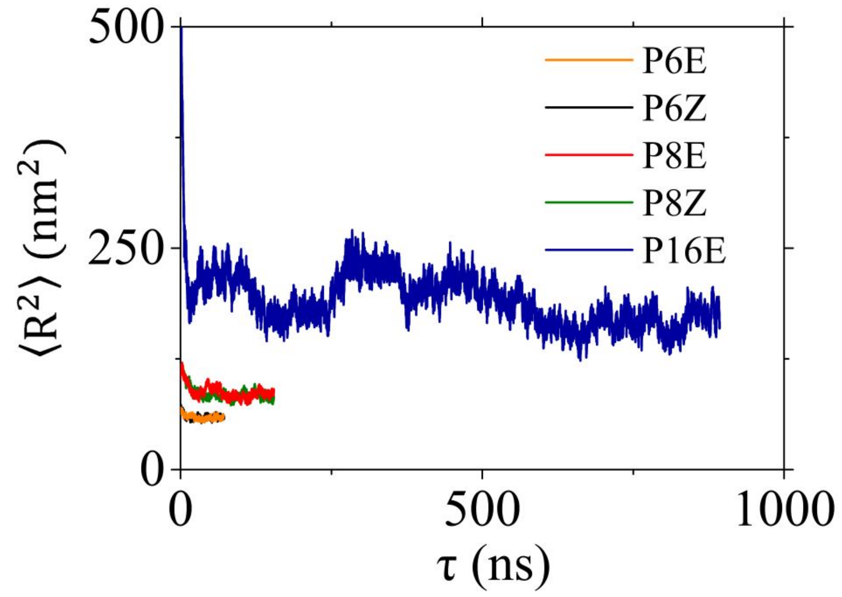

Figure S5: $\langle R^2 \rangle$ for all PCDTBT systems at T=1100K.

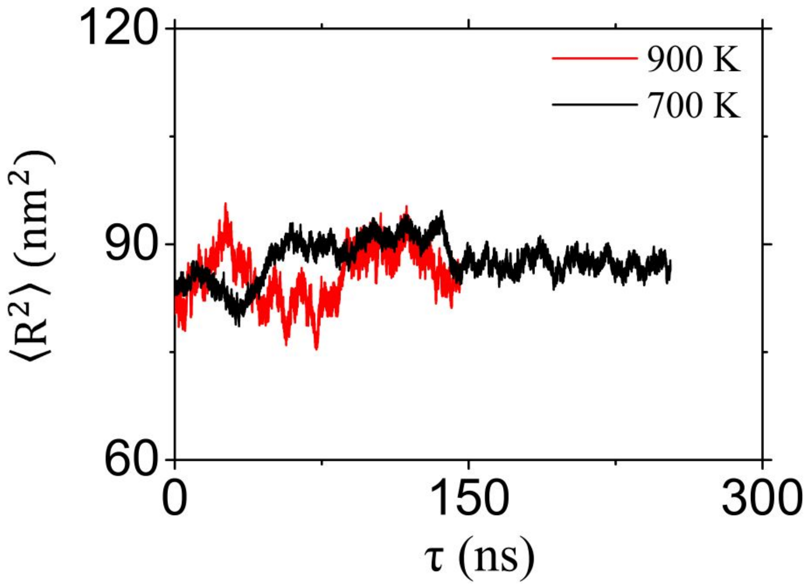

Figure S6: $\langle R^2 \rangle$ for P8Z at T= 900K and T=700K.

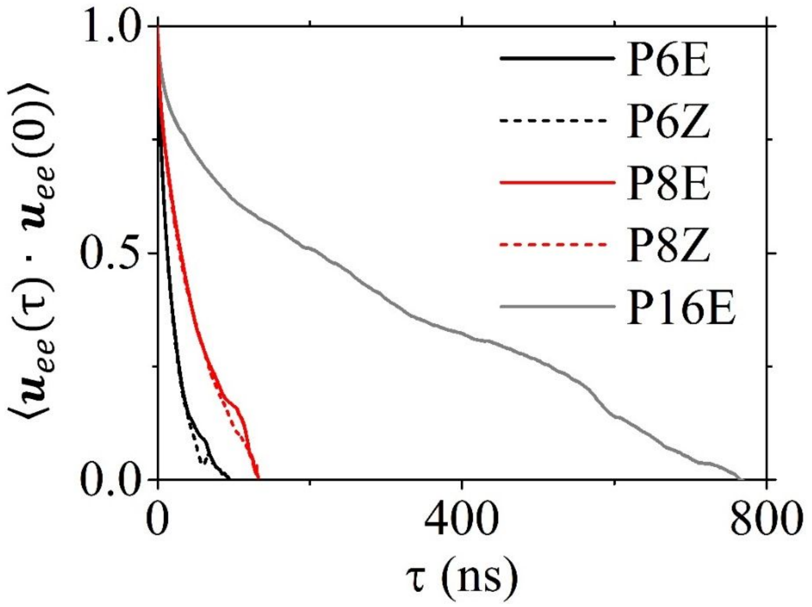

Figure S7: End-to-end TACF for all PCDTBT systems at T=1100 K.

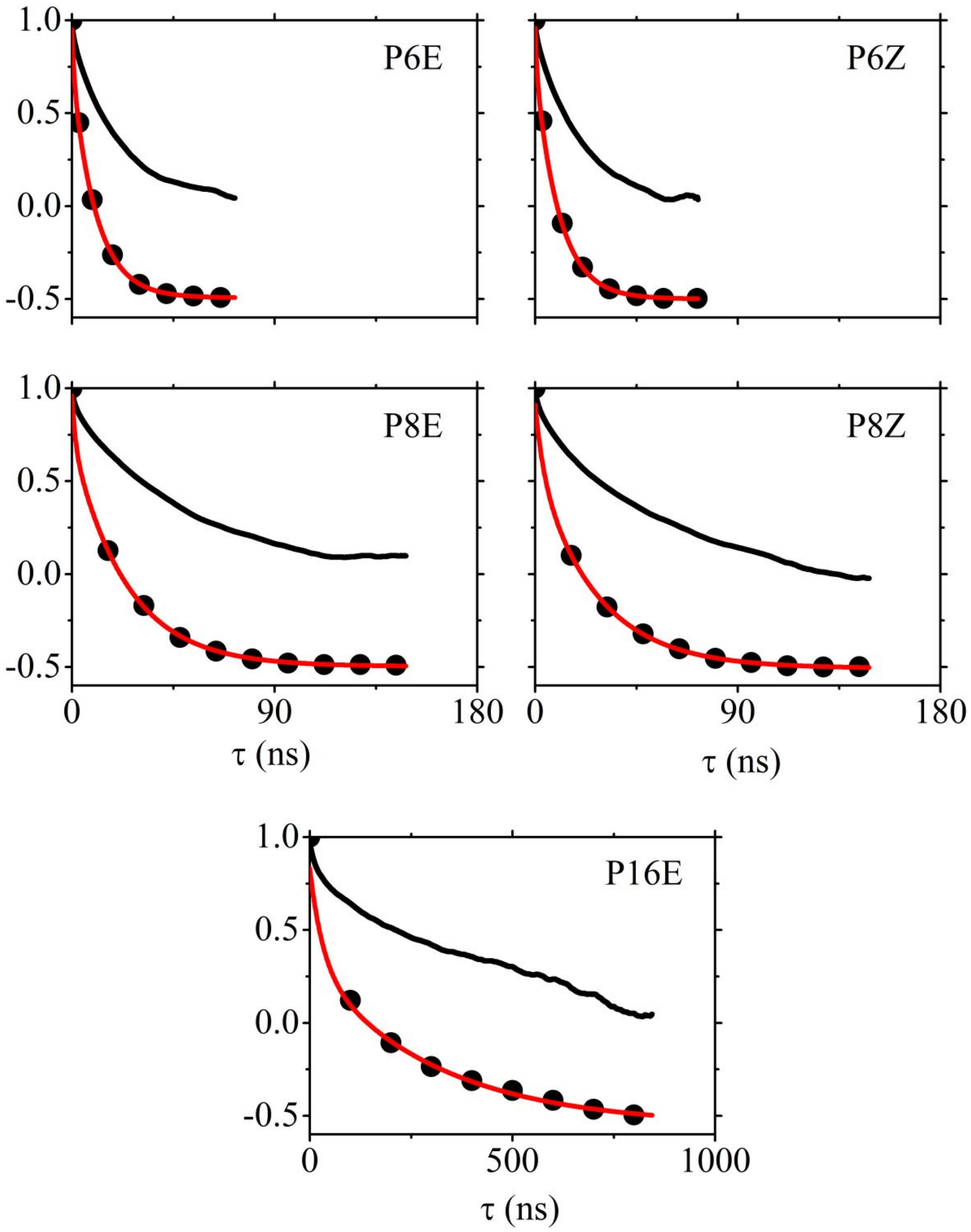

Figure S8: $\mathrm{TACF}(\tau)$ and $P_2(\mathrm{TACF}(\tau))$ for all PCDTBT systems at T = 1100 K.

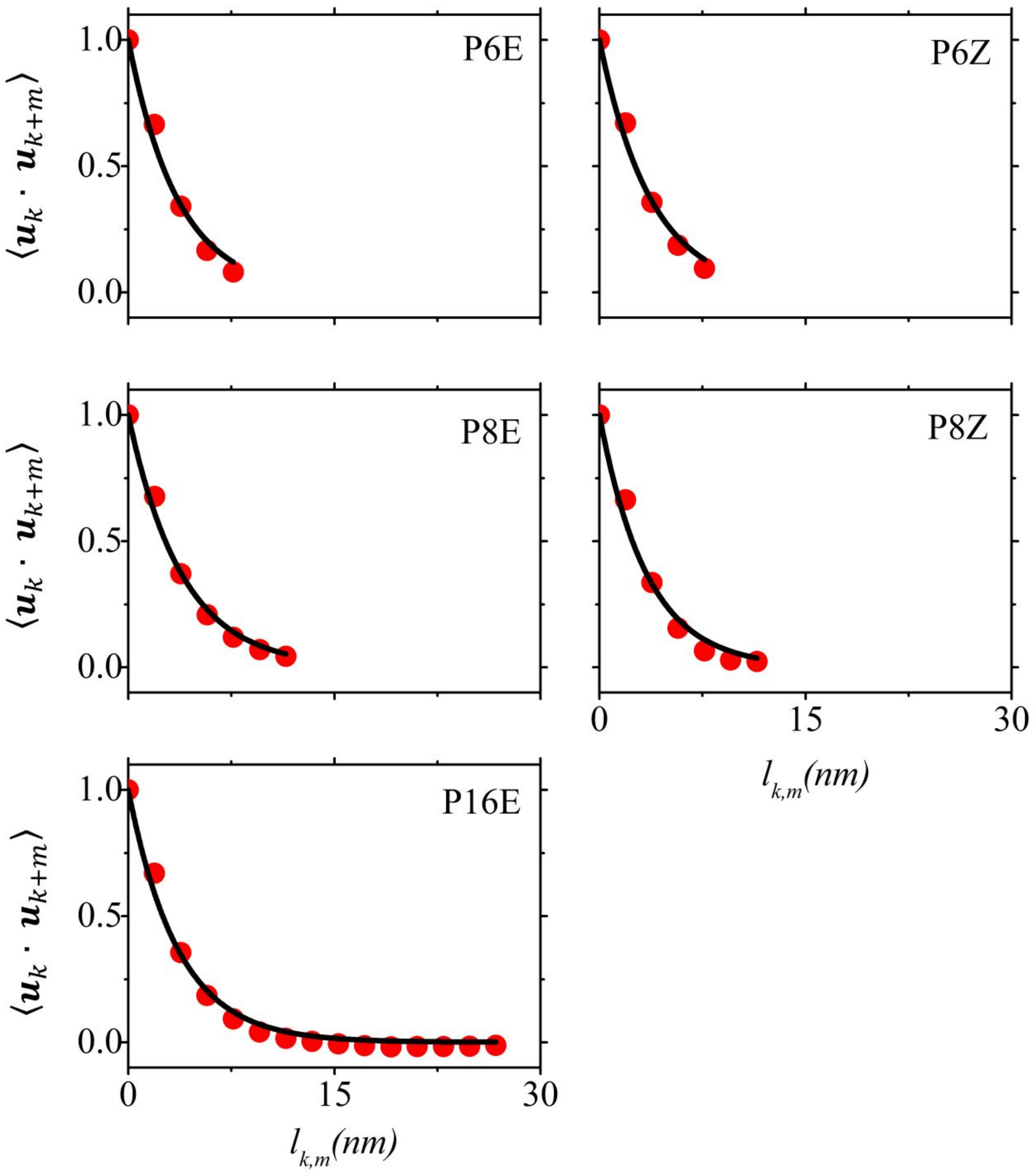

Figure S9: Autocorrelation function $\langle \boldsymbol{u}_k \cdot \boldsymbol{u}_{k+m} \rangle$ as a function of $l_{k,m}$ (red dots) and the corresponding exponential fit (black solid lines) for for all PCDTBT systems at T=1100K.

Table S1: T_c unit partial charges.

Atom	Charge
S	0.013535
C1	-0.081640
C2	-0.040952
C3	-0.219445
C4	-0.226640
H1	0.132480
H2	0.186094
H3	0.236568

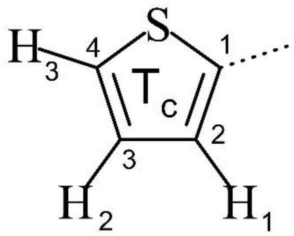

Table S2: C_i unit partial charges.

Atom	Charge
N	-0.468929
C1	-0.348673
C2	0.213710
C3	-0.293071
C4	-0.173338
C5	-0.014140
C6	-0.014140
C7	-0.173338
C8	-0.293071
C9	0.213710
C10	-0.348673
C11	0.219680
C12	0.219680
H1	0.149924
H2	0.164120
H3	0.163129
H4	0.163129
H5	0.164120
H6	0.149924

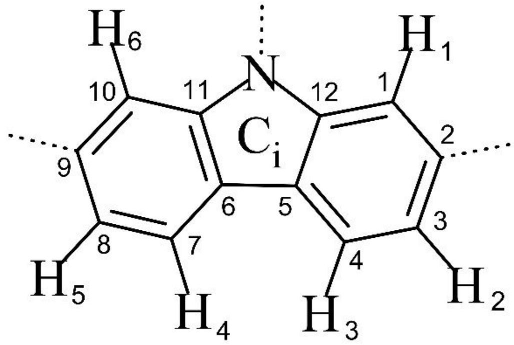

Table S3: T_i unit partial charges.

Atom	Charge
S	-0.048799
C1	0.020220
C2	-0.134253
C3	-0.149836
C4	-0.038710
H1	0.173819
H2	0.179298

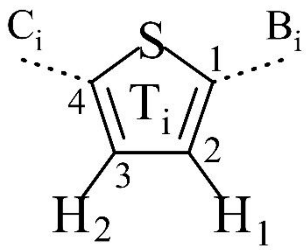

Table S4: B_i unit partial charges.

Atom	Charge
S	0.261446
N1	-0.331967
N2	-0.331967
C1	0.271837
C2	-0.078568
C3	-0.148987
C4	-0.148987
C5	-0.078568
C6	0.271837
H1	0.156962
H2	0.156962

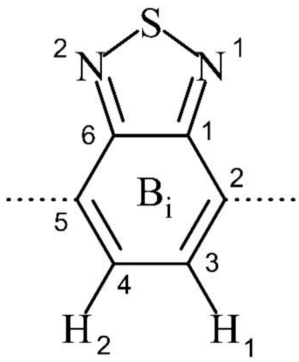

Table S5: T_b unit partial charges.

Atom	Charge
S	-0.016286
C1	-0.013812
C2	-0.033301
C3	-0.250370
C4	-0.219864
H1	0.131286
H2	0.179298
H3	0.223049

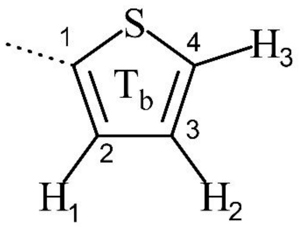

Table S6: Carbazole side chain partial charges.

Atom	Charge
C1	0.743189
C2	-0.448847
C3	0.112936
C4	0.055776
C5	0.062349
C6	-0.016258
C7	0.014557
C8	0.201266
C9	-0.298226
H1	-0.041898
H2	0.098091
H3	0.098091
H4	-0.029337
H5	-0.029337
H6	-0.014289
H7	-0.014289
H8	-0.024971
H9	-0.024971
H10	-0.012821
H11	-0.012821
H12	-0.010128
H13	-0.010128
H14	-0.038440
H15	-0.038440
H16	0.060905
H17	0.060905
H18	0.060905

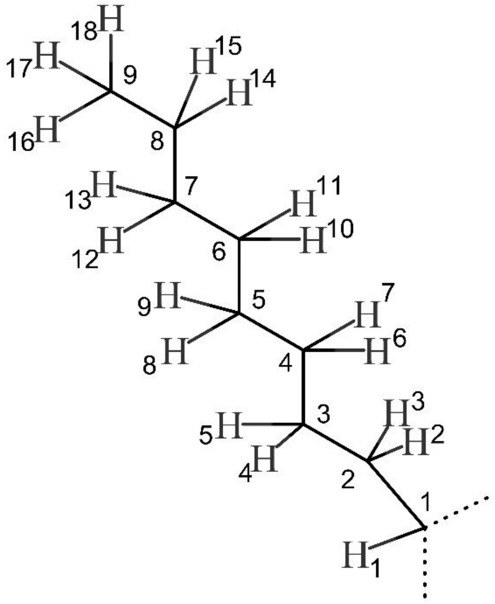

Table S7: $P_2(\mathrm{TACF}(\tau))$ relaxation times for all PCDTBT systems at T = 1100 K.

	τ_f (ns)	τ_s (ns)
P6E	1.09	10.89
P6Z	0.86	10.55
P8E	1.48	24.01
P8Z	3.51	26.52
P16E	28.69	287.20

Table S8: Persistence length (l_p) and Kuhn length (l_k) for for all PCDTBT systems at T=1100 K.

	$l_p\ (nm)$	$l_k\ (nm)$
P6E	3.61	4.61
P6Z	3.75	4.61
P8E	3.84	5.02
P8Z	3.86	4.77
P16E	4.03	5.09
